# Coping with inevitable accidents in metabolism

**DOI:** 10.1111/1751-7915.12461

**Published:** 2016-12-29

**Authors:** Antoine Danchin

**Affiliations:** ^1^ Institute of Cardiometabolism and NutritionHôpital de la Pitié‐Salpêtrière47 Boulevard de l'HôpitalParis75013France

## Abstract

Genomic studies focus on key metabolites and pathways that, despite their obvious anthropocentric design, keep being ‘predicted’, while this is only finding again what is already known. As increasingly more genomes are sequenced, this lightpost effect may account at least in part for our failure to understand the function of a continuously growing number of genes. Core metabolism often goes astray, accidentally producing a variety of unexpected compounds. Catabolism of these forgotten metabolites makes an essential part of the functions coded in metagenomes. Here, I explore the fate of a limited number of those: compounds resulting from radical reactions and molecules derived from some reactive intermediates produced during normal metabolism. I try both to update investigators with the most recent literature and to uncover old articles that may open up new research avenues in the genome exploration of metabolism. This should allow us to foresee further developments in experimental genomics and genome annotation.

## Introduction

Most of the time the cell is vibrant with chemical activity. Dormancy – the condition of the spore or the seed – is a state that lies in limbo, not dead but not quite alive. Life has to go forward. It begins with the chemical transformations which make the root of metabolism. At the ground level of life, we find dynamic transformations that extract compounds from the environment, process them into the cell's building blocks and energy supply, while discarding what is toxic or cannot be used. Continuously improved by genomic studies, a comprehensive descriptive knowledge of the matching pathways is available in textbooks and databases that provide access to all what we would like to ask about metabolism (perhaps excluding what we failed to notice). This provides us with a description of the basic chemical transformations that continuously unfold in cells. They look smooth while certainly sophisticated. Yet, as in all dynamic processes, especially those involving multiple stages, things must sometimes go awry: even metabolites must be repaired (Danchin *et al*., 2011). Despite their name, accidents are the rule, not the exception. In a famous book, Charles Perrow explored the inevitability of accidents, events that are not foreseen individually, that may be separately thought to happen extremely rarely, but that are doomed to happen sometime, and that he named for that reason ‘normal’ accidents (Perrow, 1984). In the case of the cell's metabolism, this is illustrated by the fact that molecules will inevitably be produced or modified out of the anticipated pathways. For example, metabolites become accidentally oxidized or alkylated. Furthermore, there will always be some shadow accompanying core metabolism, with reactions going off the right track, resulting in variations on the theme of normal metabolism, participating in what was named ‘underground’ metabolism (D'Ari and Casadesus, 1998) and more recently ‘paralogous’ metabolism (Chan *et al*., 2014). The latter term was proposed to illustrate the likely involvement of enzymes that were paralogues of those involved in normal metabolism as a ready‐made way to cope with metabolites that were chemical variants of the normal compounds. Many processes contribute to the way core metabolism goes astray. Rather than follow the logic of metabolism (Danchin and Sekowska, 2014), which is essentially based on a combination of chemistry, physics and geology, I explore here widely used genomic resources as well as recent articles to try and identify trends in the way cells cope with chemical accidents. Obviously, it is impossible to explore all the chemical combinations that may have some importance in the cell's life. Here, I propose some tracks that I hope will be useful to genome‐related research, via identification of solutions found by living organisms to cope with ‘normal’ accidents during the course of evolution. In order to help future genome annotators to discover unsuspected functions, I split the structure of accidents into two main topics: those resulting from radicals and those resulting from other reactive intermediates (involved in ageing and senescence in particular). In the following, I illustrate the way bacteria cope with reactive molecules with a few concrete examples based on genome studies. The way I proceed may be used for further genome functional exploration, starting, for example, from the list of reactive molecules proposed by Hanson and co‐workers (Lerma‐Ortiz *et al*., 2016). I have tried, as much as possible, to go back in time and provide reference to early work that, to my knowledge, has not been followed up but might be enlightening for future genome annotation and genome‐driven experiments.

## Unavoidable metabolic errors caused by free radicals

A first hint at possible accident‐prone reactions is the presence of so‐called spontaneous reactions (i.e. assumed to appear in the absence of an enzyme) in metabolism. The widely used reference MetaCyc knowledge base long collected such reactions. It lists at this time some 480 spontaneous reactions, basically identified by compiling articles that describe chemical pathways in the cell and require steps with apparently no associated protein. This compilation makes a fairly haphazard, but revealing collection, based on the interests of biologists, not with much background rationale: http://metacyc.org/META/search-query?type=REACTION&catal=spont (Caspi *et al*., 2016). As a consequence, a great many of those reactions correspond to quite rare compounds. I will not explore them further here, but will gather from the catalogue some reactions that have a major importance. Among spontaneous reactions, those derived from free radicals are the most obvious reactive entities produced by the cell metabolism. A first line of radical‐generating reactive species are produced in the environment, in relation with more or less random energy‐rich physical processes (photons or high energy particles coming from a variety of irradiation sources such as sunlight, cosmic rays or intrinsic radioactivity). The basal level of radioactivity (in particular in the form of ^40^K in all cells, as well as inescapable cosmic radiation) produces a low, but sustained level of free radicals. Also, chemical reactions involve electron transfers, and unpaired electrons are particularly reactive. While these processes have been the subject of a wealth of studies involving macromolecules and membranes, their effect on metabolites has seldom been explored. Furthermore, many reactions involve free radicals (the list provided in MetaCyc is sketchy, and far from complete), which are so reactive that they will find a target much faster than what enzymes would help them to do, except when they are generated in situ, and this is expected to reveal a variety of accidental targets.

Free radicals will react with almost any biological compound, resulting in chemical modifications that are subsequently recovered in the metabolite complement of the cell. While natural environmental irradiation progressively went down as the Earth came of age, the emergence of dioxygen in the atmosphere considerably enhanced radical production via reactive oxygen species (ROSs), especially in environments allowing the presence of free reduced iron. The ubiquitous Fenton reaction is a general source of the hydroxyl radical that may attack almost any of the cell's components (Anglada *et al*., 2015). In parallel, related reactive nitrogen species (NO^•^ in particular (Pullan *et al*., 2008)) and reactive sulfur species (Giles and Jacob, 2002) contributed to the general free radical burden of the cell. It is important to note, though, that this should not be considered as always negative: for example, evolution recruited free radicals to be used by cells as means to compete invading organisms (Frawley and Fang, 2014). Hence, metabolism is generally replete with free radicals as well as a plethora of non‐radical but highly reactive derivatives (singlet oxygen, hydrogen peroxide or hypochlorite, which easily generate radical ROSs) that will contribute to the panel of unconventional metabolites in the cell.

A first line of defence for the cell is to act before these reactive entities are generated, via destruction of some of their sources. To this aim, it uses specific enzymes (peroxiredoxins, catalase, superoxide dismutase, glutathione peroxidase, in particular). In the case of reactive nitrogen species, NO may enter the nitrogen cycle (Eady *et al*., 2016). In a second line of defence, cells evolved tolerance processes (Giuffre *et al*., 2014). Some organisms, such as Lactobacilli and Mycoplasmas, have alleviated the bulk of dioxygen‐dependent radicals generation by dispensing of iron and using manganese as a ROS scavenger (Lisher and Giedroc, 2013). When annotating genome sequences, it is essential to have this countermeasure in mind as it will impact overall genome annotation (Danchin and Fang, 2016). A general shielding process (that I do not discuss here as this would ask for another full article) is for the cell to protect reactive groups such as amines or thiols using available metabolic resources. This might account for widespread methylation, acetylation or succinylation of amine, imidazole, guanidinium or thiol groups (Levitan *et al*., 2010; Lu *et al*., 2011). Some of these modifications would have subsequently be recruited for regulation (and phosphorylation appears mainly to be used as a regulatory tag) and a general exploration of protection/deprotection processes in the cell should be undertaken (see (Chan *et al*., 2014)), in particular because the very protection systems (S‐adenosylmethionine in particular) may accidentally modify unwanted targets. To escape unavoidable metabolic accidents, a general way out is for cells to use the buffering capacity of some of its components, as I now discuss.

### Sulfur building blocks: cysteine and methionine

As an example of the way cells cope with these events, I illustrate the situation created by the presence of the sulfur atom, which is a particularly reactive nucleophile. When sulfur‐bearing targets have been modified, a repair mechanism can operate, preventing accumulation of an altered cell building block which might enter paralogous metabolism. A typical example is provided by alkylation of cysteines in proteins, which after proteolysis will generate S‐alkyl‐cysteines, analogues of proteogenic amino acids that are therefore toxic and need to be put aside (Chan *et al*., 2014). ROS will also react with cysteine residues, which, via their chemical reactivity, manage redox states, first by creating and disrupting disulfide bonds. Subsequently, oxidation may go to the unstable sulfenic (‐S‐OH, (Gupta and Carroll, 2014)), then sulfinic (‐S‐O_2_H) and finally sulfonic (‐SO_3_H) form of the amino acid (van Bergen *et al*., 2014). The sulfenic form is fairly easily reduced back to ‐SH via pathways involving glutathione (or related molecules, such as *N*‐acetyl‐cysteine or bacillithiol in Firmicutes and mycothiol in Actinobacteria, and sometimes coenzyme A), thioredoxin and other enzymes involved in thiol management. The SufL(YajL) chaperone forms mixed disulphides with target proteins, preferentially at sulfenylated cysteines (Gautier *et al*., 2012). This sulfur oxidized form is also an entry point in the biosynthesis of a likely ROS buffer, ergothioneine, made by fungi and bacteria (Song *et al*., 2015), opening up a new track for oxidized sulfur metabolism.

When it happens (this appears to be rare in Bacteria, but ought to be explored in‐depth), reduction in the subsequent oxidation stage, sulfinic acid, requires a stronger reducing power, and enzymes of the energy‐dependent sulfiredoxin family, acting mainly on oxidized peroxiredoxin (Boileau *et al*., 2011). Because these modifications are pervasive they have been recruited by bacteria as sensors of ROSs and other reactive species beside their role as buffers against their deleterious action. Finally, when oxidation results in formation of cysteic acid, the modification is irreversible and the protein must be degraded. Subsequently, cysteic acid may be exported out of the cell or used directly, or indirectly after decarboxylation into taurine, as a sulfur source via desulfation (Cook *et al*., 1998). Coenzyme A, which is not always protected by carrying an acyl‐group, is also likely to suffer similar accidents. Almost nothing is known about the fate of such CoA derivatives except for it common disulfide form (Wallace *et al*., 2012) and the highest oxidation state, which is likely to be catabolized as other sulfonates (Nakamura and Tamura, 1972).

**Figure 1:**
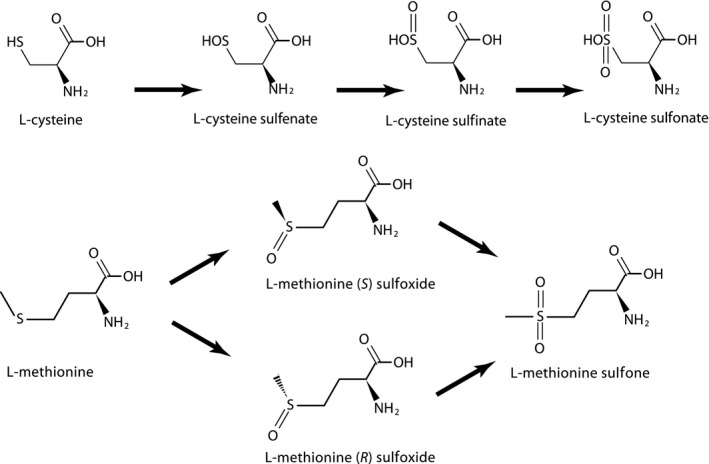
Progressive oxidation of cysteine and methionine residues.

Beside oxidation of cysteine, two diastereoisomers of methionine sulfoxide, (*S*)‐ and (*R*)‐ may be formed in oxidized proteins, depending on the radical and on the environment (Davies, 2005). Remarkably, to cope with this widespread situation, cells evolved two geometrically symmetrical catalytic sites, stemming from completely different protein descents but displaying mirror symmetry of their catalytic site, methionine sulfoxide reductase A and B (Achilli *et al*., 2015). There is even another class of these enzymes, belonging to molybdoenzyme families, MsrPQ (see also below), for repairing proteins found in the periplasm of diderm bacteria (Gennaris *et al*., 2015). Yet, despite the existence of such ROS reversible ‘sponge’‐like behaviour of methionine, it is expected that some oxidized proteins will be degraded, releasing free methionine sulfoxides in the cell. Furthermore, free methionine can be oxidized directly as well. Now, methionine sulfoxide is an analogue of glutamine (Chin and Means, 1996) that could enter translation, and this needs to be dealt with. In fact, the structural similarity between oxidized methionines and glutamine may explain why glutamine tRNA synthetase did not spread to all bacterial clades. The existence of a likely ancient homeotopic modification (Danchin, 1989) of glutamate by an amidotransferase on glu~tRNAgln in many bacterial clades (e.g. Firmicutes (Strauch *et al*., 1988)) may be accounted for by avoidance of misacylation of tRNAgln by oxidized methionine. More generally, cells cope with this oxidation using reductases that act on free methionine sulfoxides. In Enterobacteria, for example, an enzyme that repairs oxidized biotin but not protein oxidized methionine, biotin sulfoxide reductase BisC (Denkel *et al*., 2013), is sufficient for growth on methionine‐(*S*)‐sulfoxide (Ezraty *et al*., 2005). Further, another member of the Msr family (fRMsr), MsrC(YebR) (Etienne *et al*., 2003; Lin *et al*., 2007), is present in *Escherichia coli* extracts and reduces the other diastereoisomer, free methionine‐(*R*)‐sulfoxide, to methionine. Subsequently, oxidation could go further to reach methionine sulfone (Fig. 1), a compound that is fairly stable and cannot be used as a methionine substitute in animals or a great many bacteria (Or‐Rashid *et al*., 2003). Specific peptidases allow release of free methionine sulfone that is then excreted in the environment (Payne and Tuffnell, 1981). The very fact that the environment is not flooded by this compound demonstrates that, at least for some microorganisms, methionine sulfone could be degraded and presumably used as a sulfur source. We have to go back in time to find work that identified some of the corresponding catabolism (Tonzetich, 1976).

A similar situation is met with oxidized derivatives of tryptophan or histidine, for example, but for the sake of space, I do not further explore here the way cells cope with these metabolic alterations (see (Lin *et al*., 2016) for cell management of 2‐oxohistidine peptides), noting, however, that this is of considerable interest for microbiome studies (Kennedy *et al*., 2016).

### Nucleic acids: the example of purines

By contrast, to show how our present knowledge could be used in similar contexts, I dwell some more on derivatives of nucleotides, as they play a central role in energy management, mutagenesis and regulation, with many genes involved in the proofreading and maintenance processes. The list of the corresponding metabolites is far too long to be described here more than in a sketchy way, be it only because RNA nucleotides can have a considerable number of modifications (Machnicka *et al*., 2015). Remarkably, almost nothing is known about the fate of these nucleotides when they are released in the cell after RNA (or, to a smaller extent, DNA) degradation, which happens often concomitantly with inactivation of the corresponding molecules by reactive chemical species, radicals in particular. Now, again, the environment would be replete with these molecules if there did not exist fairly efficient degradation pathways: we certainly can expect that, among the many genes of unknown function that keep being discovered as more genome and metagenome sequences are deciphered, we will find enzymes involved in the corresponding processes.

As a case in point, here is the fate of some purine derivatives. Guanine derivatives (as related purine derivatives) are very sensitive to ROS, in reactions similar to: GTP + hydroxyl radical → 8‐oxo‐GTP + H^+^ (this also happens within nucleic acids in particular when G is unpaired). When input in DNA, 8‐oxoG (a tautomer of 8‐hydroxy‐guanine, 6,8‐dihydroxy‐2‐amino‐purine) will be mutagenic, producing transversions, and when in messenger RNA, producing missense proteins. Beside some examples of reactions involving 8‐oxoG, the MetaCyc database lists a small fraction of the consequences of OH^•^ reactivity (alteration of nucleic acids via production of 8‐oxo‐guanine, 2‐hydroxy‐adenine (isoguanine) or 5‐hydroxy‐cytosine, but also 5‐hydroxyuridine). All produce metabolites that must be exported or degraded by the cell. The pathways of nucleotide degradation should provide a rich set of novel activities. The cell protects itself first by preventing 8‐oxoG assimilation into nucleic acids, via degradation of 8‐oxo‐(d)GTP into 8‐oxo‐(d)GMP [using the MutT enzyme, a member of the Nudix hydrolase family (Hamm *et al*., 2016)]. Enzymes of this family will cope with situations (in particular alkylation of nucleotides) when a nucleotide analogue must be prevented to cross the genome sequence quality control, by hydrolysing pyrophosphate from the triphosphate group of the nucleoside triphosphate analogue. Interestingly, quite a few proteins of this family do not yet have a firmly established function (see, e.g. in *E. coli* for modified pyrimidines, NudG(YnjG) and NudI(YfaO)).

At this point, it becomes important to depart from the current practice, that assumes that functional conservation implies structural similarity, to adopt a functional view (function first, structure second) in order to try and identify enzymes with the expected functions. We know that Firmicutes and Proteobacteria, for example, recruited quite different structures to cope with a variety of functions associated with replication (Engelen *et al*., 2012). As a consequence, it should not be surprising that degradation of nucleotides differs between these clades. For example, there is no structural counterpart of *E. coli* guanine deaminase GuaD in *Bacillus subtilis*. Yet, an enzyme with guanine deaminase activity, GuaN, is present in the latter. However, this time, it belongs to the cytidine (hence a pyrimidine) deaminase superfamily (Liaw *et al*., 2004). Interestingly, using our Phyloprofile algorithm (Engelen *et al*., 2012), we can see that GuaN is co‐evolving with xanthine dehydrogenase and a variety of functions related to nitrogen metabolism. Furthermore, it co‐evolves with methionine sulfoxide reductases and oxygenases, substantiating indeed that it is involved in catabolism of oxidized purine derivatives (Table S1). This observation opens up another whole domain of possible structures that can cope with modified nucleotides, nucleosides and nucleic acid bases. Furthermore, of course, this does not preclude the possible existence of other families of structures with similar functions. Remarkably, GuaN co‐evolves with a considerable number of proteins with no ascribed function (‘Y’ proteins, Table S1), suggesting that exploration of the *Bacillus* clade would surely be rewarding in terms of identification of novel catabolic activities.

In parallel, in *E. coli* YrfG preferentially hydrolyses phosphate in purine nucleotides. It could also be used to prevent other modified purines to get into nucleic acids. To be sure, a variety of modified nucleotides could be degraded by proteins of the same family, with YjjG preferring pyrimidines, whereas YieH hydrolysed both purines and pyrimidines as secondary substrates (Kuznetsova *et al*., 2006). The corresponding family of haloacid dehalogenase‐like enzymes should be studied in priority to decipher the large number of expected activities that will be essential to bacterial communities. Indeed, the frequent production of halogenated metabolites in sea environments has been proposed to be related to metabolic accidents (Manley, 2002).

**Figure 2:**
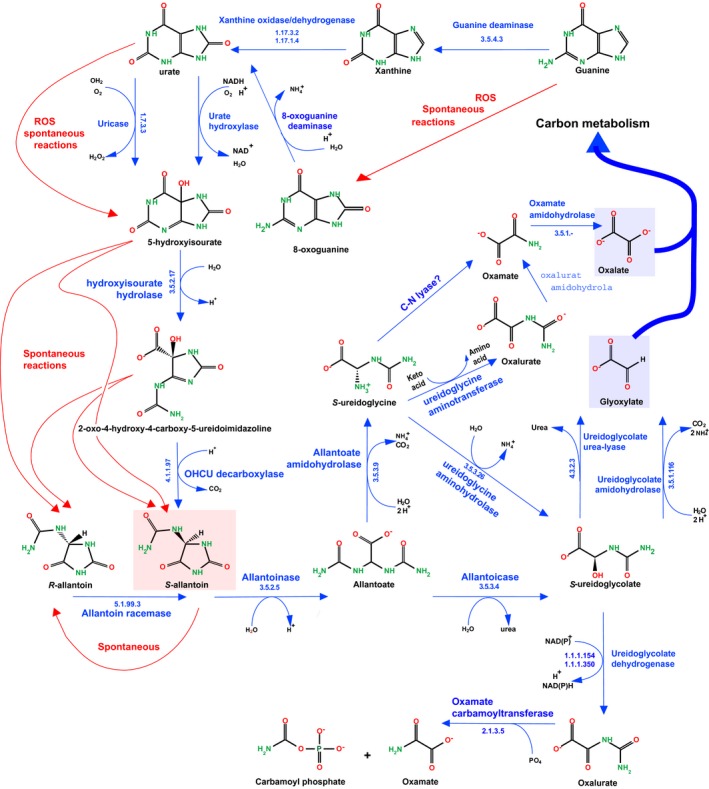
Degradation of guanine and 8‐oxoguanine. Spontaneous reactions are as indicated, showing how specific enzymes were sometimes recruited to prevent them to occur.

Subsequently, as an expected straightforward functional pathway, a nucleosidase (phosphorylases might also play a similar but energy saving role, as in the methionine salvage pathway (Sekowska *et al*., 2004)) would liberate 8‐oxoguanine. In *E. coli,* deaminase GuaD may, beside guanine, accept 8‐oxoguanine as substrate. However, this implies some promiscuity, which may lead to unwanted reactions so that the existence of a specific deaminase seems likely. Interestingly, the deamination of 8‐oxoG would directly form uric acid, an entry point into fairly involved but ubiquitous degradation pathways (described in some details below, Fig. 2). It was therefore predicted that this specific deaminase should exist, at least in some organisms. Finding the enzyme proved difficult. It was eventually identified in *Pseudomonas aeruginosa* (Hall *et al*., 2010b). Exploration of metagenome data derived from environmental DNA sequences isolated from bacteria in the Sargasso Sea under the Global Ocean Sampling Project identified further amidohydrolases of the 8‐oxoguanine deaminase family, catalysing the deamination of isoxanthopterin and pterin‐6‐carboxylate (Hall *et al*., 2010a). Guanine and pterins are both metabolically and structurally related, and this is witnessed by promiscuous reactions involving both types of metabolites (Kim *et al*., 2009). This makes obvious that there is a wide class of enzymes with related functions that may act on pterin or guanine oxidized or nitrosated derivatives. Unknown function protein SsnA in *E. coli,* for example, belongs to the COG0402 family and has the conserved signature PxxVxTHxHxxQ present in amidohydrolases and its gene belongs to an island that may be involved in modified nucleic acid bases break down.

Following guanine deamination, xanthine oxidase (XO) oxidizes hypoxanthine. It also acts on other purines and on pterins and a variety of aldehydes. Under some conditions, it may produce superoxide rather than peroxide. The *E. coli* enzyme XdhABC is an FAD/molybdopterin‐dependent dehydrogenase that uses NAD^+^ as a cofactor (EC 1.17.1.4, xanthine dehydrogenase). In diverse organisms, the enzyme can be converted to an O_2_‐dependent XO form (EC 1.17.3.2). Other XO‐type enzymes such as *E. coli* aldehyde dehydrogenase PaoABC harbour an additional [4Fe‐4S] cluster. They have a scavenging function acting on aromatic aldehydes, not purines, and act as periplasmic detoxifying enzymes involved in oxidation of aldehydes, possibly on a variety of plant‐derived aromatics, but also possibly on some of the modified nucleic acid bases (Correia *et al*., 2016). Catabolism of aromatics is particularly significant in Pseudomonadales that, beside carbon cycles, use a variety of enzymes to degrade heterocycles. In general, their authentic substrates have not yet been deciphered (see (Belda *et al*., 2016), and the corresponding regularly updated knowledge base at https://www.genoscope.cns.fr/agc/microscope/mage/viewer.php?O_id=2834). Future metabolomic studies should help identify some of the pathways that evolved to cope with unavoidable accumulation of modified nucleic acid degradation products.

The subsequent fate of urate is remarkably diverse. Some of it is illustrated in Fig. 2. Remembering that annotations in databases are often unreliable (Percudani *et al*., 2013), it is essential to try and follow the chemical constraints in the postulated pathways and reappraise current annotations. In aerobic cells, urate may be oxidized to 5‐hydroxyisourate by two distinct enzymes: either a coenzyme‐independent urate oxidase (uricase, EC 1.7.3.3) found in eukaryotes and bacteria like *B. subtilis* (PucL) or a prokaryotic flavoprotein urate hydroxylase (HpxO) originally found in some *Klebsiella* species (Michiel *et al*., 2013). The *B. subtilis* enzyme is fused at its N‐terminal end to a domain similar to *Mycobacterium tuberculosis* AhpG, an alkyl hydroxyperoxide reductase that cleaves reactive oxygen intermediates. This activity would remove hydrogen peroxide formed in the uricase‐catalysed oxidation of uric acid to allantoin. Next, PucL catalyses oxidation of uric acid to 5‐hydroxyisourate (HIU) to *S*‐allantoin and possibly also the stereoselective decarboxylation of 2‐oxo‐4‐hydroxy‐4‐carboxy‐5‐ureidoimidazoline (OHCU), created by hydrolase PucK (Schultz *et al*., 2001). Still another related spontaneous reaction has been suggested: uric acid acting as an antioxidant in the periplasm may produce 5‐hydroxyisourate, so that periplasmic HiuH(YedX), that hydrolyses 5‐hydroxyisourate, would belong to the arsenal protecting the cell from free radicals. In *E. coli* and other diderms, HiuH thus produces OHCU which spontaneously decays into a racemic mixture of *R*‐ and *S*‐allantoin. Furthermore, non‐enzymatic decomposition of both HIU and OHCU does occur and does so non‐stereospecifically. On top of this, another spontaneous reaction, non‐enzymatic racemization of allantoin has been observed. Genomes will tend to recruit enzymes that could cope with the outcome of such spontaneous reactions or channel them along specific pathways. To be sure, two types of allantoinase have been identified, a metal‐dependent form and a metal‐independent form. While these two enzymes may differ in mechanism, they both preferentially bind the (*S*)‐enantiomer of allantoin. This suggests that HiuH may also have OHCU decarboxylase activity, although this is not straightforward. Alternatively, or in complement, there may exist an allantoin racemase, as indeed discovered in *Klebsiella pneumoniae* (French *et al*., 2011).

Subsequently, allantoin can be degraded via a variety of pathways, also with intermediates that are unstable and interconvert spontaneously, yielding potentially compounds that will be hazardous to the cell metabolism (Fig. 2). As a consequence, a number of enzyme activities were recruited in the different environments of living organisms to cope with this possibly harmful situation. Oftentimes, degradation proceeds via ureidoglycine and/or ureidoglycolate and ends up with glyoxylate, urea and/or ammonium, allowing cells to recover carbon and nitrogen. Another intermediate, oxamate, was identified as one of the end‐products of allantoin degradation in *Streptococcus allantoicus,* and further studies demonstrated that oxamate formation is catalysed by an oxamate transcarbamylase possibly via formation of oxalurate as in *B. subtilis* (via ureidoglycine aminotransferase) or *E. coli*. To our knowledge, a direct pathway from ureidoglycine to oxamate is not known. In this context, protein YgeW has a 3D structure related to arginine, ornithine and putrescine carbamoyltransferases, but belonging to a distinct clade, which is indicative of a novel activity. It has been suggested to be an oxamate carbamoyltransferases (transcarbamylase) involved in purine catabolism. However, oxamate, allantoin and other substrates were tested but purified YgeW had no activity on any of them. Yet it might generate oxamate in a *S*‐ureidoglycine degradation pathway: ureidoglycine could not be tested in the assays if the protein precluding identification of this activity if it was indeed present (Li *et al*., 2011). Subsequently, oxamate would release oxalate and ammonium, as observed for oxamate amidohydrolase HpxW in *Klebsiella michiganensis* (Hicks and Ealick, 2016). To substantiate this view, I note that, using the phyloprofile software in *B. subtilis* shows that protein YwrD is very similar to the *Klebsiella* HpxW counterpart and that it co‐evolves with most of the enzymes involved in the xanthine degradation pathway (Table S2).

Remarkably, periplasmic degradation of purines is functionally associated in *E. coli* with MsrPQ, another set of molybdoenzymes – consistent with their co‐evolution (Medigue *et al*., 2005), that I have previously described. The *hiuHmsrPQ* operon is under the control of the two‐component system MsrVW(YedVW) which appears to monitor ROSs (Gennaris *et al*., 2015) and chlorine (Melnyk *et al*., 2015) species, in addition to excess copper (Urano *et al*., 2015). Curiously, while this protection process is acknowledged as an important feature, its explicit consequences are not displayed in‐depth in most studies. As can be seen from what is described here, a large variety of metabolic pathways could cope with purine degradation. When investigating the metabolism of organisms that are far from model organisms, it is therefore important to avoid directly transposing previous knowledge, but, rather, to proceed via functional analysis, looking for necessary functions first. In general, assuming catabolic pathways as already known may be very misleading, in particular in systems biology studies (Chavarria *et al*., 2013).

## Other reactive intermediates

In a study quite remarkable for its implications and that should belong to the library of all those interested in metabolism, the laboratory of Andrew Hanson recently summarized the role of spontaneous chemistry in the cell's context. There are so many possibilities, however, that the authors had to make a choice among their preferred molecules. They established a list of 30 top compounds subject to accidents, prominent in metabolomic studies (Lerma‐Ortiz *et al*., 2016). In contrast to the MetaCyc list, which is essentially based on literature, the Chemical‐Damage‐MINE database repository of reactive compounds http://minedatabase.mcs.anl.gov/#/top30/S1 is presented in a chemically consistent manner. As a consequence of their potential to disrupt the cell's functions, we may expect that these reactive compounds will require agents (essentially enzymes, hence genes) able to remedy the effects of their reactivity, correcting errors in metabolism while redirecting unwanted compounds back to the core pathways. This should be revealed by genome studies. A well‐known example is hydrolysis of 6‐phosphogluconolactone, long believed to undergo rapid spontaneous hydrolysis, but which would react with intracellular nucleophiles, and which is therefore metabolized by a lactonase (Miclet *et al*., 2001). I only investigated further here two families of reactive molecules because they are likely to be important during the process of cell ageing, a process that has only been sketchily studied in bacteria: those involving RidA and RidA‐related activities and those involving glycation and related processes.

### Enamine/imine deaminases/hydrolases

Enzyme catalysis removes the obstacle of high energy of activation in specific reactions via a variety of intermediary steps, involving highly reactive compounds, often as the result of the action of a coenzyme. Pyridoxal phosphate, in particular, will produce molecules with reactive double bonds, which under normal circumstances easily react with a specific substrate, ending up in the normal product of the reaction. Yet, in particular during the many transitions (e.g. temperature) faced by living organisms, the reactive intermediate may diffuse out of its enzyme, possibly meeting neighbouring substrates. In transamination, racemization or deamination reactions, reactive amino acid derivatives such as aminoacrylate/iminopropionate will be produced. The laboratory of Diana Downs has identified the RidA protein in *Salmonella enterica* (formerly YjgF/YabJ/UK114, often still annotated as a ribonuclease) as the major enzyme for scavenging these intermediates (Lambrecht *et al*., 2013).

The *E. coli* genome contains five counterparts of RidA, paralogs TdcF, RutC(YcdK), YoaB and YjgH. TdcF is likely to be a direct counterpart of RidA, preferably acting on four carbon metabolites (associated with anaerobic threonine degradation pathway) instead of three (related to serine via 2‐aminoacrylate). It may be important in processes that prevent the accumulation of l‐*allo*‐threonine in the cell. RutC is involved in a catabolic pathway of pyrimidines that produces 3‐aminoacrylate (Kim *et al*., 2010). YoaB and YjgH evolved together, as well as with RutC (Table S3). Furthermore, the synteny with *ridA*(*yjgF*) around *yjgH* is often conserved. This suggests that the corresponding activities are perhaps overlapping, dealing with deamination of enamine/imines produced at intermediary steps in a variety of metabolic pathways. The role of these proteins may be linked to changes in osmolarity, as co‐evolving genes involved in osmolarity control are co‐evolving with them, while a link with serine deamination is noticeable in the hydroxypyruvate co‐evolving genes (Table S3 and Fig. 1 in de Lorenzo *et al*., 2015). Hanson *et al*. also noticed that *ridA* counterparts are often clustered with genes involved in carbamoyl‐phosphate metabolism, coding for an unknown activity that future work will understand (Lerma‐Ortiz *et al*., 2016). Finally, a remarkable observation may account for diversification of the corresponding protein sequences: RidA appears to metamorphose into a molecular chaperone when modified by N‐chlorination (Muller *et al*., 2015). The role of these enzymes is still far from completely understood. It links metabolism of branched‐chain amino acids, serine and threonine and pyrimidines in a network that is not yet fully deciphered. Derepressibility of the *ilvGEDA* operon in *E. coli* is a common control point, which is still used as an easy characterization of *relA* mutations (a *relA* mutant does not grow on a minimal medium supplemented with either serine, methionine, glycine or uracil, methionine, leucine, 1 mM each, unless supplemented with isoleucine (Uzan and Danchin, 1978)). Involvement of these enzymes of core processes of life ought to be explored in priority.

### Carbonyl species and related >C=O containing molecules

Carbon metabolism in cells produces a large number of reactive aldehydes and ketones. These compounds are reactive carbonyl species (RCS), critical cell‐damaging and signalling agents. To those, we may associate the less reactive alpha‐keto acids, among which we find the precursors of amino acids and their degradation products. Briefly, transamination characterizes anabolism while decarboxylation associated with formation of thioesters marks catabolism. I do not discuss this further, except to stress again that the reaction of these compounds with ammonia or primary amines forms imine derivatives, making Schiff bases and RidA‐like enzymes important to cope with their reactivity (Lerma‐Ortiz *et al*., 2016). In the reactive carbonyl functional group, >C=O, the carbon atom has two remaining bonds that may be occupied by hydrogen, alkyl or aryl substituents. In carboxylates, this electron distribution is shared between the carbon and the two oxygen atoms, making the group considerably less reactive (unless it can be associated with a conjugated double bond or as bicarbonate in specific environments). Some consequences of their reactivity are now explored.

#### Reducing sugars

Carbohydrates that can convert into an open chain with a terminal aldehyde group act as reducing compounds that react with free amines (Maillard reaction, initially discovered at high temperature). This happens quite frequently in the cell, depending on the environment of the amine group, resulting in glycated proteins. The carbonyl group of the sugar often reacts with the amino group of the amino acid, producing N‐substituted glycosylamine and water. The initial stages of the reaction occur more rapidly with fructose than with glucose, making fructose derivatives particularly frequent but by no means the only glycated compounds that matter. Glycated proteins are often non‐functional and prone to degradation or to aggregation. They further evolve into advanced glycation end‐products (AGE) proteins after Amadori reactions that impair proteolytic digestibility and alter protein conformation. While this process is ubiquitous (Mironova *et al*., 2001; Kram and Finkel, 2015), it has mainly been studied in human being, because of its pathogenic consequences (Ajith and Vinodkumar, 2016) and because Amadori compounds are produced during cooking, a process which is associated with a specific human ability, domestication of fire. The most frequent adducts are fructose‐lysine as well as similar adducts of the N‐terminal amino acid. They may be repaired in proteins by specific deglycases (Table 1) such as previously discussed SufL(YajL) in *E. coli*, a glyoxalase that has also deglycase activity (Abdallah *et al*., 2016). SufL converts alpha‐oxoaldehydes to carboxylic acids. Its overexpression enhances cellular protection from exogenously added glyoxals and reduces the glyoxal‐dependent increase in intracellular AGEs. As noticed here previously, it also acts as a covalent chaperone for sulfenylated thiol proteins involved in Fe‐S construction and maintenance.

**Table 1:** Protective metabolism against methylglyoxal and glyoxal

*Escherichia coli*			*Bacillus subtilis*		
Name	Activity	PubMed	Name	Activity	PubMed
MgsA	Methylglyoxal synthase	19458924, 21831320	MgsA	Methylglyoxal synthase	21992469, 23894131
GloA	Glyoxalase I; hemithioacetal‐glutathione isomerase, lactoyl‐glutathione forming	25670698	GlxA YwbC	Glyoxalase I; hemithioacetal‐bacillithiol isomerase, lactoyl‐bacillithiol forming	24330391, 25283443
GloB YafR	Lactoyl‐glutathione hydrolase	25670698	GlxB YurT	Methylglyoxalase; lactoyl‐bacillithiol hydrolase	24330391, 25283443
HchA YedU	Deglycase; glyoxalase III and molecular chaperone Hsp31	26774339, 26678554	—		
ElbB	Oxoaldehyde oxidase; glyoxalase	9603997, 26678554	—		
GlxO YhbO	Glyoxalase O; oxoaldehyde oxidase; deglycase	26678554, 26774339	SufLB YfkM	General stress protein 18; deglycase	24330391, 25283443
SufL YajL ThiJ	Oxoaldehyde oxidase; glyoxalase; chaperone, protecting proteins in response to oxidative stress; deglycase	26678554, 26774339, 27644758	SufL YraA	Deglycase; general stress protecting enzyme; protects against methylglyoxal toxicity	24330391, 26774339
DkgA YqhE	Beta‐keto ester reductase; 2,5‐diketo‐d‐gluconate reductase; MGO reductase	20676725, 25108218	YvgN YvsB	Promiscuous GO/MGO reductase active *in vitro* on glyceraldehyde and glyceraldehyde‐3‐phosphate	19585557
YqhD	1,2‐propanediol dehydrogenase; alcohol dehydrogenase; aldehyde dehydrogenase (NAD(P)‐dependent)	20543070	YugJ YugK	Duplicated butanol dehydrogenase (verified in *Clostridium acetobutylicum*)	1809209
DkgB YafB	2,5‐diketo‐d‐gluconate reductase; 4‐nitrobenzaldehyde reductase; MGO reductase	22328670	YtbE	Promiscuous aldo/keto reductase preferential specificity for derivatives of benzaldehyde	19585557
YdjG	Methylglyoxal reductase (NADH‐dependent)	16077126, 22074179	AkrN YhdN	NADPH‐dependent aldo/keto reductase; protects against methylglyoxal	24330391
Gpr YghZ	l‐glyceraldehyde 3‐phosphate reductase (NADPH‐dependent); MGO reductase; GO and glycolaldehyde reductase	20015532,23990306	—		
YajO	2‐carboxybenzaldehyde reductase; deletion increases MGO metabolites	23990306, 25326299	YrpG	Aldo‐keto reductase; deletion does not change MGO sensitivity	24330391
YqiI	Fimbrial protein involved in detoxification of methylglyoxal	17846588	—		
KefGKefB	Potassium proton antiporter; activated by lactoyl‐glutathione	7934942, 21143325	—		
KhtL YabL	Proton/potassium antiporter; activated by glutathione (by inference)	7934942, 24603618	KhtU YhaU	Proton/potassium antiporter; methylglyoxal resistance; activated by bacillithiol	23894131, 24330391

Because of widespread glycation, glycated‐amino acids generated after proteolysis are ubiquitous compounds that can be used by many bacteria as carbon or nitrogen sources. For example, in *B. subtilis,* the operon *frlBOMND* codes for transport and metabolism of fructose‐amino acids (Deppe *et al*., 2011). A counterpart exists in *E. coli*. Fructoselysine in *E. coli* is phosphorylated by a specific kinase (FrlD), followed by the conversion of fructoselysine‐6‐phosphate into glucose 6‐phosphate and lysine by fructoselysine‐6‐phosphate deglycase (FrlB). There is considerable variation on this theme, depending on the species (Gemayel *et al*., 2007). Several bacterial operons comprise a homologue of fructoselysine‐6‐phosphate deglycase, an homologue of the isomerase domain of glucosamine‐6‐phosphate synthase, and components of a phosphotransferase system (PTS), presumably transporting the fructose‐amino acids, but often with no FrlD homologue (Wiame *et al*., 2005). In *S. enterica*, deglycases GfrE and GfrF are required for growth on glucoselysine and fructoselysine respectively. Experimental observations are consistent with a pathway in which fructoselysine and glucoselysine are phosphorylated at the C‐6 position of the sugar by the GfrABCD PTS as they are transported across the membrane (Miller *et al*., 2015). Fructosamine kinases are active in the intracellular environment, where they produce 3‐deoxyglucosone, which promotes AGE formation because it is seldom metabolized before it interacts with a variety of targets. Because these modifications are frequent in cooked food it is interesting to observe that human commensals may use them as major growth supplies (Bui *et al*., 2015).

Amadoriases, also known as fructosyl amino acid oxidases (FAODs) or fructosyl amine oxidases (FAOXs), are enzymes found in fungi and bacteria. These enzymes catalyse the de‐glycosylation of fructosyl amino acid to yield a free amine, glucosone and hydrogen peroxide. They have been categorized into three groups, depending on their substrate specificity. Group I FAOX are active on alpha‐fructosyl amino acids (amino acids glycated on backbone amines), and group II FAOX are active on epsilon‐fructosyl amino acids (amino acids glycated on side‐chain amines), while group III FAOX show similar activity on either alpha‐ or epsilon‐fructosyl amino acids (Rigoldi *et al*., 2016). In *E. coli*, enzymes such as SolA (acting *in vitro* on sarcosine and other secondary amino acids), which is similar to *Aspergillus fumigatus* amadoriase, may display this activity. Indeed, its co‐evolution profile suggests that its function is related to a large number of transport systems and to metabolism of possibly toxic aldehydes, sulfur metabolism and degradation of oxidized molecules (Table S4).

#### Methylglyoxal and other aldehydes and ketones

One of the most prominent characters of dicarbonyls is their high electrophilicity. Among these compounds, methylglyoxal (MGO) is a major endogenous toxic metabolite. It makes covalent bonds with nucleophilic groups (e.g. found in arginine, cysteine and lysine residues in proteins) (Rabbani and Thornalley, 2012). Glyoxal (GO) is a counterpart that displays similar toxicity but is part of an active process that may limit its production (glyoxylate shunt (Jahan *et al*., 2016)). The toxic behaviour of MGO is the more remarkable as a great many organisms have specific enzymes (e.g. in *E. coli* MgsA(YccG) methylglyoxal synthase, EC 4.2.3.3, also present in human beings) meant to synthesize it from dihydroxyacetone phosphate (Kosmachevskaya *et al*., 2015). Conservation of this function implies that some element in the cognate metabolism is so important that it is maintained despite the toxicity of the molecule. A common hypothesis to account for this somewhat puzzling observation is that the pathway leading to MGO is used for ATP‐independent glucose catabolism. It could thus be linked to phosphate homoeostasis. In *B. subtilis*, Crh, an homologue of PTS phophocarrier protein HPr, appears to control MgsA synthesis, linking its role to carbon catabolite repression and phosphate transfer (Landmann *et al*., 2011). A further hypothesis is that while shunting the last stages of glycolysis, the pathway would limit NADH and possibly serine production, which has harmful consequences (de Lorenzo *et al*., 2015).

MGO and GO reactions with amino acids produce a variety of adducts resulting in advanced glycation products (Kaur *et al*., 2016). They also react with nucleic acids (Thornalley, 2003). As a consequence, a considerable number of enzymes have evolved activities inactivating these molecules, whether as their primary target, or via moonlighting. In *E. coli,* for example, we find an arsenal of enzymes that act directly or indirectly on GO and MGO, as well as on a variety of aldehydes, also reactive by themselves (Table 1). A major, fairly widespread, detoxification pathway comprises two steps, involving GloA, glyoxalase I (with a Ni^2+^ cofactor) that forms (*R*)‐S‐lactoylglutathione from MGO and glutathione, and GloB(YafR), glyoxalase II, which is an hydroxyacylglutathione hydrolase producing d‐lactate and regenerating glutathione. This pathway is very general, and conserved in *B. subtilis* (GlxA/GlxB), despite the considerable phylogenetic distance of this species from *E. coli*. However, there it uses bacillithiol as cofactor instead of glutathione. Further, a different set of enzymes does not use reduced sulfur as cofactor: in *E. coli*, HchA(YedU) is a stationary phase glyoxalase III that catalyses a cofactor‐independent conversion of MGO to d‐lactate. It also repairs MGO‐ and GO‐glycated proteins, releasing repaired proteins and lactate or glycolate respectively (Abdallah *et al*., 2016). Another enzyme, ElbB, possibly involved in isoprene biosynthesis, has also moonlighting activity on glycated proteins. Finally, the conserved oxoaldehyde oxidase SufL (YraA in *B. subtilis*), again, has already been discussed. In *E. coli*, it has a counterpart, glyoxalase O (GlxO(YhbO), YfkM in *B. subtilis*), that converts alpha‐oxoaldehydes to carboxylic acids. It seems also to have deglycase properties. Overexpression of these enzymes enhances cellular protection from exogenously added GOs and reduces the GO‐dependent increase in intracellular AGEs. Note that the *B. subtilis* enzymes are not authentic orthologs of their *E. coli* counterparts, as they depend on bacillithiol not glutathione. In the same way, counterparts in Mycobacteria are expected to use mycothiol instead of glutathione. However, all these enzymes arose from a common descent and a relevant nomenclature has to be designed to take this cofactor variation into account.

In line with a role of MGO synthesis in the balance between reduced and oxidized NAD cofactors, DkgA is a NADPH‐dependent beta‐keto ester reductase/2,5‐diketo‐d‐gluconate reductase that also displays GO/MGO reductase activity. Its paralog DkgB(YafB) is primarily a 2,5‐diketo‐d‐gluconate reductase that reduces 4‐nitrobenzaldehyde *in vitro* and acts as a MGO reductase. These enzymes appear to be conserved in *E. coli* and *B. subtilis* (Table 1). YdjG (AkrN(YhdN) in *B. subtilis*) is a further aldehyde reductase, coded in a carbohydrate degradation operon of yet unknown specificity, that also acts as a NADH‐dependent MGO reductase. Several other enzymes acting on aldehydes may also efficiently detoxify GO, among which Gpr(YghZ) l‐glyceraldehyde 3‐phosphate reductase/MGO reductase, and YeaE are major NADPH‐dependent players (Lee *et al*., 2013). Ultimately, general purpose oxidoreductase proteins such as AldA behave as NADH‐dependent aldehyde dehydrogenases that act on glycolaldehyde and lactaldehyde, thus minimizing their toxicity. A great many paralogs of this enzyme are present in most organisms. Finally, in *E. coli,* YqiI is a fimbrial protein somehow involved in detoxification of MGO, possibly as a buffering element (Table 1). Overall cells display a large supply of oxidoreductases with considerable buffering role on aldehydes, and in particular GO and MGO. It is, however, expected that because these metabolites are pervasive, these reactive metabolites will, as time elapses, contribute significantly to protein ageing. A thorough study of the contribution of these enzymes that are probably redundant via moonlighting, should be undertaken.

An additional defence mechanism is worth emphasizing. It has been mainly deciphered in *E. coli* and *B. subtilis* (Chandrangsu *et al*., 2014), but is likely to operate in a great many other organisms as well. MGO toxicity is highly sensitive to pH. A slight acidification of the cell's cytoplasm is enough to reduce the number of MGO targets (Ferguson *et al*., 1993). As a consequence, cells developed a proton‐based protection system where potassium is exchanged for protons during efflux. This is substantiated by the observation that the (*R*)‐S‐lactoyl‐glutathione intermediate in MGO detoxification is required for the activation of the KefGB proton/potassium efflux pump (Ozyamak *et al*., 2010). Finally, in *B. subtilis*, the chromosome neighbourhood of the *mgsA* gene, coding for MGO synthase, is revealing. It comprises the *bshA* and *bshBA* genes, coding for two enzymes required for bacillithiol synthesis, embedded within a seven‐gene operon additionally including *mgsA*, and the essential genes *cca* and *birA*, encoding tRNA nucleotidyltransferase (CCA transferase) and biotin‐protein ligase respectively (Gaballa *et al*., 2013). To be sure, mutants of this system are more sensitive to MGO.

#### Fumarate

While authentic dicarbonyls are usually highly reactive and toxic, carboxylates are generally fairly innocuous because of their low spontaneous reactivity. However, the presence of a conjugated carbon double bond such as that in fumaric acid makes this molecule quite reactive. Indeed, the tricarboxylic acid cycle enzyme fumarate hydratase, that inactivates this reaction trend, has been identified as a tumour suppressor in a subset of human renal cell carcinomas. Among other possible targets, lysine in particular, cysteine residues in proteins are especially prone to react with fumarate, forming S‐(*2*‐succinyl)cysteine (Alderson *et al*., 2006). Subsequently, upon protein degradation, this metabolite is likely to accumulate in cells and either be exported or degraded via a still unknown pathway. This reaction that appears to be commonplace in a variety of human diseases (in particular associated with mitochondria, including obesity and diabetes (Thomas *et al*., 2012)), is likely to be ubiquitous. Remarkably, it is associated, in Parkinson's disease, with modification of a detoxification family of enzymes that I found in the previous paragraph to be involved in protection against protein glycation (Poschmann *et al*., 2014). Taken together, these observations show that fumarate reactivity should be revisited carefully in bacterial genomes exploration. Furthermore, because protein degradation would generate unusual non‐proteinogenic amino acids, that do not accumulate in the environment, unknown catabolic pathways are likely to be uncovered in the future.

#### Carboxylation

In the cell's internal medium, carbon dioxide is generally present in the hydrated form of bicarbonate. Due to the essential role of the carbon dioxide/bicarbonate buffer system in regulation of physiological pH, CO(3)(•‐), it may be one of the most important ROS in biological systems, and it may contribute to formation of 8‐oxoG, discussed previously (Roginskaya *et al*., 2015). Furthermore, as shown by the process of light‐dependent carbon fixation, as well as carboxylation of pyruvate in the main anaplerotic reaction in carbon metabolism, carbon dioxide is certainly not devoid of reactivity. As a matter of fact, the addition of a carboxyl group to the epsilon‐amino group of the lysine side chain is a frequent but widely overlooked post‐translational modification with remarkable consequences. Lysine is positively charged at physiological pH. However, the carboxyl group inverts the electrochemical properties of the lysine side chain by complementing the roles of glutamate and aspartate in the fine tuning of enzyme activity. Providing a negative charge instead of a positive one on a long side group expands the catalytic capabilities of the amino acids in proteins beyond the 20 common amino acids. Carboxylated lysine is the structural counterpart to arginine, except that its side chain is negatively charged at the pH range useful for enzymes. Experiments with urease demonstrated that this modification occurs spontaneously under basic pH conditions involving carbon dioxide (Park and Hausinger, 1995) and without the mediation of any enzyme. Witnessing the importance of this modification, the class D beta‐lactamases are characterized by the presence of a carboxylated lysine in the active site that participates in catalysis (Che *et al*., 2014). Despite its likely significance, the extent of lysine carboxylation in proteins is currently unknown. A lysine carboxylation software has been designed. Its outcome running on a variety of conditions suggests that about 1.3% of large proteins may contain a carboxylated lysine residue (Jimenez‐Morales *et al*., 2014). This amply demonstrates the importance of putting some focus on this type of post‐translational modifications.

## Conclusion

As new genome sequences keep accumulating, the number of genes with no ascribed functions increases steadily. This is such a large amount that we cannot expect to be able to set up experimental approaches that will allow us to identify their function. Identification of gene function is somewhat similar to criminal profiling (Verde and Nurra, 2010). We have to use hypotheses, data and context to try and propose a candidate. In this quest, we have three ways to go forward: deduction, which uses a preset hypothesis imposed upon the physiological, biochemical and genetic observations associated with the corresponding gene and deduce what it must be. Induction, that proceeds the other way around, tries to infer a function by association. Finally, abduction, a shot‐in‐the‐dark approach (you carry a gun, while in a forest, and fire: if something cries, you go there and see what you got…), applies a harsh treatment to the data and looks for the way it behaves. The most efficient way forward is induction. Yet most genomic studies tend to unknowingly use abduction, with a considerable amount of spurious observations that plague the future of predictions. The design of knowledge bases constructed on the concept of neighbourhoods (Nitschke *et al*., 1998) must be further developed to improve our ability to make valid inferences. I have tried here to create a picture of some relevant neighbourhoods. I hoped to show how this approach might be used in future investigations of ‘omics’ approaches meant to sort out the fate of normal accidents in metabolism.

## Conflict of interest

None declared.

## Supporting information


**Table S1.** Co‐evolution pattern of *B. subtilis* guanine deaminase GuaN. The protein co‐evolves with enzymes involved in purine degradation. Note the very large number of proteins of unknown function (213/500 “Y” proteins) suggesting that exploration of cognate pathways in Firmicutes will be rewarding.

**Table S2.** Co‐evolution pattern of *B. subtilis* amidohydrolase YwrD. Note that the protein evolves with most of the enzymes involved in purine catabolism. Many of the 141 remaining “Y” proteins have a related activity that can be identified. Nomenclature should be modified accordingly.

**Table S3.** Functional co‐evolution of YjgH in *E. coli*. The protein co‐evolves with RutC and YoaB. Many proteins of the 136 remaining “Y” proteins have an identified activity, showing that the nomenclature needs to be modified urgently. Furthermore those associated to oxygenase activities should perhaps be studied in priority.

**Table S4.** Co‐evolution pattern of *E. coli* SolA protein. SolA may be an amadoriase. It is associated to co‐evolution of 163 “Y” proteins, many of them putative transporters. In addition several are putative phosphatases, that may correspond to phosphorylated carbohydrate derivatives.
